# Exploring the lack of continuity of care in older cancer patients under China's ‘integrated health system’ reform

**DOI:** 10.1093/ageing/afae213

**Published:** 2024-10-07

**Authors:** Jiawei Geng, Ran Li, Xinyu Wang, Rongfang Xu, Jibing Liu, Dixi Zhu, Gaoren Wang, Therese Hesketh

**Affiliations:** Centre for Global Health, School of Public Health, Zhejiang University, Hangzhou, China; Institute of Oncology, Affiliated Tumour Hospital of Nantong University, Nantong, China; Centre for Global Health, School of Public Health, Zhejiang University, Hangzhou, China; Insititue of Global Health, University College London, London, UK; School of Public Health, Nantong University, Nantong, China; Department of Nursing, Affiliated Tumour Hospital of Nantong University, Nantong, China; Institute of Oncology, Affiliated Tumour Hospital of Nantong University, Nantong, China; Department of Health Management, HanYao Traditional Chinese Medicine Hospital, Nantong, China; Institute of Oncology, Affiliated Tumour Hospital of Nantong University, Nantong, China; Centre for Global Health, School of Public Health, Zhejiang University, Hangzhou, China; Insititue of Global Health, University College London, London, UK

**Keywords:** continuity of care, primary care, older patients, care coordination, cancer, qualitative research, older people

## Abstract

**Background:**

Continuity of care is essential to older patients’ health outcomes, especially for those with complex needs. It is a key function of primary healthcare. Despite China's policy efforts to promote continuity of care and an integrated healthcare system, primary healthcare centres (PHCs) are generally very underused.

**Objectives:**

To explore the experience and perception of continuity of care in older cancer patients, and to examine how PHCs play a role in the continuity of care within the healthcare system in China.

**Methods:**

A qualitative study using semi-structured interviews was conducted in two tertiary hospitals in Nantong city, Jiangsu province, China. A combination of deductive and inductive analysis was conducted thematically.

**Results:**

Interviews with 29 patients highlighted three key themes: no guidance for patients in connecting with different levels of doctors, unmet patients’ needs under specialist-led follow-up care, and poor coordination and communication across healthcare levels. This study clearly illustrated patients’ lack of personal awareness and experience of care continuity, a key issue despite China’s drive for an integrated healthcare system.

**Conclusion:**

The need for continuity of care at each stage of cancer care is largely unmeasured in the current healthcare system for older patients. PHCs offer benefits which include convenience, less burdened doctors with more time, and lower out-of-pocket payment compared to tertiary hospitals, especially for patients with long-term healthcare needs. However, addressing barriers such as the absence of integrated medical records and unclear roles of PHCs are needed to improve the crucial role of PHCs in continuity of care.

## Key Points

- Older cancer patients reported no continuity of care, a key issue despite China’s push for an integrated healthcare system.
- The lack of gatekeeping and minimal coordination between professionals are major barriers to involving PHCs in care continuity.
- Healthcare financing plays an important role in ensuring continuity of care.
- Older patients often struggle with multimorbidity, facing inconsistent treatment and unclear information during transitions.
- The inherent advantages of PHCs, especially for older cancer patients, are proximity, avoiding travel inconvenience.

## Background

Continuity of care is defined as the degree to which a series of healthcare events is experienced as coherent, connected, and consistent with the patient's medical needs and personal context [1]. The World Health Organization (WHO) identifies four domains of continuity of care: interpersonal continuity, longitudinal continuity, management continuity and information continuity [2]. High-quality continuity and coordination of care are crucial to health outcomes [3, 4] and should be key functions of primary care where people generally make first contact with the health systems [5]. However, continuity of care has received relatively little attention in many countries, especially in most low-and-middle-income countries, including China [6, 7].

China has a three-tier system for healthcare delivery: health facilities and providers operate at county, township and village levels in rural areas, and at municipal, district and community levels in urban areas. Primary healthcare centres (PHCs), including county, township hospitals and community healthcare centres, are providers of primary care services in China [8]. The primary role of PHCs in China has focused on the prevention and monitoring of common chronic diseases and infectious diseases, as well as health education [9].

Public hospitals in China have received <10% of their annual funding from the government since 2000, resulting in perverse incentives that force hospitals to adopt a pay-for-services (FFS) system to generate revenue [10]. Consultation costs are low at all levels in China, and with no gate-keeping role for primary care, patients often bypass lower levels of care and go directly to tertiary hospitals [11]. As a result, there is a serious imbalance between huge demand at tertiary hospitals and underutilisation of PHCs in much of China, with consequent inefficiency in use of resources [12]. Despite this, the rapid growth of an ageing population with complex multi-morbidities has made the role of primary care much more relevant, especially given that PHC doctors are less burdened than their counterparts in larger hospitals [13].

In recognition of these challenges, China has implemented a series of policies aimed at improving continuity of care and redressing the primary-tertiary care imbalance. These policies include increasing investment from both central and local governments in PHCs; offering a 10% higher reimbursement rate under the national health insurance system for medical costs incurred at PHCs; and most importantly, establishing medical alliances since 2016 [14, 15]. These alliances, also known as the ‘integrated healthcare system’ are designed to integrate tertiary hospitals, secondary hospitals and PHCs into a unit with two-way referral and remote medical consultation within a specific area, usually district or county. Tertiary hospitals within these alliances are tasked with training lower-level facilities and improving their clinical capability [16]. By 2021, >15 000 medical alliances had been established in 205 cities or counties [17].

Although resources have been allocated to specifically strengthen the primary care system, the planned integration has been slow to develop in many areas [18]. There is no financial connection between tertiary hospitals and PHCs within one medical alliance, many PHCs are managed by tertiary hospitals but are rarely used by tertiary hospital physicians for follow-up care [19]. Based on recent results from the China Health and Retirement Longitudinal Survey, utilisation of PHCs as a percentage of the total number of visits to healthcare facilities has shown a decreasing trend from 2010 to the present [20].

Cancer is one of the most complex diseases of older adults and is well-known to benefit from continuity of care approaches [21]. In 2020 ~10 million adults over the age of 65 were living with cancer globally [22]. Overall, ~24% of the world’s cancer diagnoses and 30% of the world’s cancer deaths took place in China in 2020 [23]. As recommended in the ‘integrated healthcare system’ policy, cancer patients should register with PHCs which have responsibility for routine check-ups and follow-up care, guided by oncologists [24].

According to a systematic review conducted in 2023, which examined the financial burden for cancer patients in China, the mean medical costs ranged from USD 7421 to USD 10 297 per patient during the first 12 months after a cancer diagnosis, with out-of-pocket expenditures ranging between USD 4875 and USD 5020 [25]. In comparison, the average medical costs ranged from USD 12 361 to USD 23 343 per cancer patient in the initial 12 months in Germany, with an out-of-pocket expenditure of no more than USD 2570 [26].

To date, almost all the existing studies about continuity of care for cancer patients have been in high-income countries with well-established primary healthcare systems. Globally, the quantitative evidence shows that continuity of care is linked to lower healthcare resource utilisation and improved health outcomes along cancer care pathways [27–29]. Qualitative studies from the UK have highlighted the importance of effective communication, care coordination and emotional support in ensuring continuity of care for cancer patients [30, 31]. To our knowledge, there is no qualitative research exploring older cancer patients’ experiences and perceptions of continuity of care in China.

## Research aims and objectives

We conducted this qualitative study to gain insights into the extent to which patients had experienced continuity of care, as well as to reflect on how PHCs within the new integrated system can be involved in the longer-term care of older cancer patients in China. The objectives were: (i) to explore patients’ experiences of continuity of care during hospitalisation and aftercare; (ii) to identify factors that had impacts on patients’ experience; (iii) to investigate patients’ underlying preferences for continuity of care. This study focuses on continuity of care for older cancer patients to identify general implications for continuity of care under the integrated healthcare system.

## Methods

### Study design

This qualitative study involved interviews with patients over the age of 65 after in-hospital cancer treatment. To present the findings, the Consolidated Criteria for Reporting Qualitative Studies (COREQ) checklist was followed (Appendix 1) [32]. Ethical approval was obtained from the Ethics Committee of School of Public Health Zhejiang University (ZGL202101-5), and the Ethics committee of Nantong Oncology Hospital (TZL-2023031).

### Study settings and participants

This study was conducted in Nantong city, Jiangsu province, China, a city with a total population of 7 million, of which 20% are over 65 years of age, the highest proportion in China. Nantong was designated as a pilot city for the ‘integrated healthcare system’ reform, and by 2022, a total of 70% of over-65s were being managed for chronic conditions such as hypertension and diabetes in PHCs [33]. However, the need for CoC for cancer patients, who require long-term health management, has not been prioritised.

Participants were identified from a questionnaire survey regarding satisfaction of healthcare services, administered to all cancer patients hospitalised from March to December 2022 in Nantong Oncology Hospital and the cancer department of the Affiliated Hospital of Nantong University. These two tertiary hospitals provide services to ~80% of cancer patients in the city.

At the end of the questionnaire, patients were asked if they were willing to participate in an interview about their experience and understanding of continuity of care. A total of 42 patients expressed interest in participating. The target population was patients aged 65 years and over, following in-hospital treatment for cancer, including chemotherapy, radiotherapy and surgical treatment. The types of cancer were lung, gastric, colon, liver, breast and prostate, the most common cancers in older adults in China [34]. Thirteen patients with terminal cancer or any cognitive or mental disorder were excluded.

### Data collection

A purposive sampling method was used to obtain a sample of patients by age, sex and cancer type. Family members were allowed to be present and join the interview, if requested by the patient. We had planned to conduct all interviews face-to-face. However, the restrictions of COVID-19 made this impossible. Therefore, we conducted interviews with 17 patients by phone at their home and 12 patients face-to-face on the ward, just prior to discharge, between July 2022 and January 2023. Interviews lasted 40 min on average.

Interview guides (Appendix 2) were structured following the WHO continuity and coordination of care framework mentioned above. To help patients clarify their experience, perceptions, concerns, constraints and contexts of their healthcare experience, we divided the interview guides into three phases: before admission, at discharge, and post discharge and longer-term care. We started analysing data after the first three interviews were conducted, so the qualitative process was iterative, with responses informing the subsequent interviews; new questions were added, and some questions were simplified. Interviews were discontinued when saturation was reached.

### Data analysis

**Figure 1:**
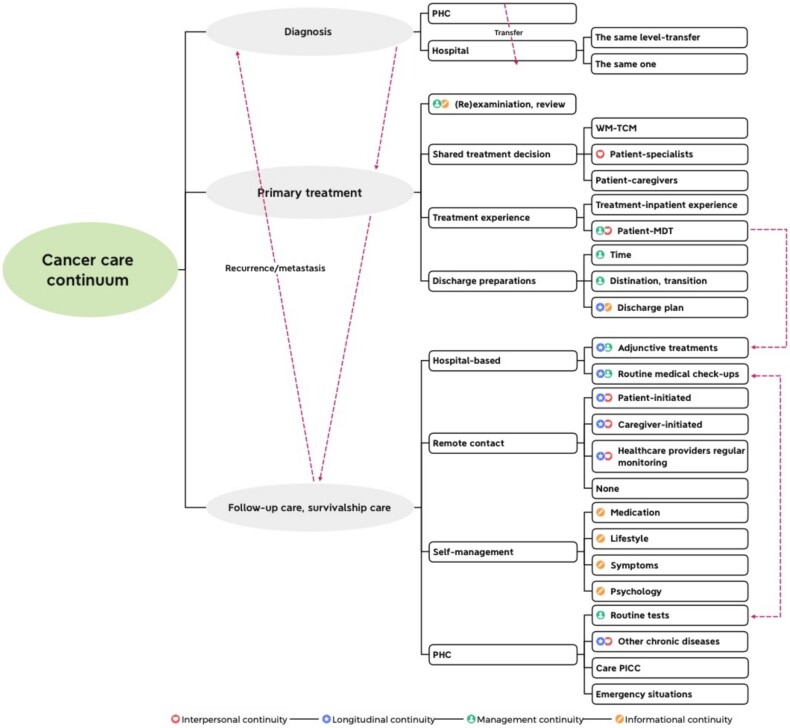
Code tree of emerging items during interview.

All interviews were recorded and transcribed verbatim. The initial translation of the transcriptions from Chinese to English was performed by a bilingual researcher. Subsequently, another researcher back translated the English transcriptions into Chinese to ensure accuracy. Any misunderstandings or unclear wording identified during the back-translation process were resolved by consulting an English native speaker to better reflect the nuances of the target language. A combination of deductive and inductive thematic analysis was conducted to generate a thematic framework of patients’ experiences and perceptions of continuity of care. Two researchers analysed data using QSR NVIVO 12 software. After reading and familiarisation with transcripts, we open-coded each interview independently to capture emerging items. Codes were based on small text fragments, forming a code tree (Figure 1), which was adjusted multiple times during the process. When divergence arose, we discussed these with an expert (TH) who has extensive experience in qualitative research on healthcare systems and patients’ health seeking behaviour in China to achieve consensus. Initial codes were then generated and grouped according to their similarity. We independently classified quotations corresponding to each category. The categories were organised into sub-themes. Then, we identified the linkages between categories and sub-themes with different aspects of continuity of care to create the thematic framework. All data have been anonymised.

## Results

In total, we completed interviews with 29 patients, 14 women and 15 men between the ages of 65 and 82, covering six cancer types and different stages of treatment, including after surgery, chemotherapy and radiotherapy. The time since diagnosis ranged from 1 week to 10 years. Of total participants, 18 patients were from the Oncology Hospital and the remaining 11 were from the Affiliated Hospital of Nantong University. The characteristics are shown in Appendix 3.

**Figure 2:**
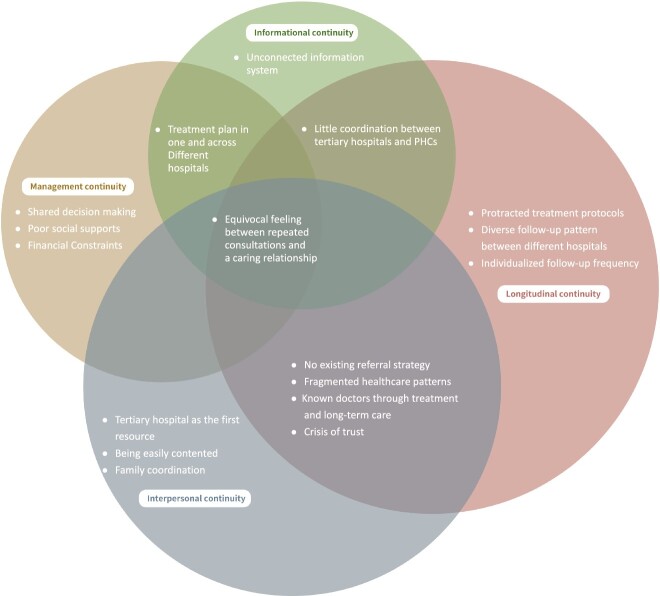
Factors, patients’ preferences and underlying issues in four domains against the WHO guideline of continuity of care.

It is important to note that none of the patients interviewed, actually described any experience of ‘continuity of care’ despite explanation. Therefore, we identified three themes that encapsulated the lack of continuity of care throughout medical treatment and aftercare. Under each theme, we summarised the system-related and patients’ preference-related factors that impacted on patients’ experience of the lack of continuity of cancer care (Table 1). We also analysed the patients’ views of involving PHCs in continuity of care, as recommended, under the current healthcare model. As shown in Figure 2, the factors and underlying issues were categorised into the WHO four domains for continuity of care. They were mapped as overlapping, where it merged across two or more domains.

**Table 1:** Themes and subthemes.

	Theme	Subtheme	
		System-related factors	Patients’ preferences-related factors
Patients’ experience of the lack of continuity of cancer care			
	Before admission: Absence of a named doctor as a navigator	No existing referral strategy	Tertiary hospital as the first resource
	During treatment: discrepancies in information exchange between providers and patients	Poor coordination during the treatment phase Financial constraints	Conflicts in decision making
	Post-discharge: dilemmas concerning patients’ needs and specialist-led follow-up care	Overburdened tertiary doctors Fragmented healthcare patterns	Individualised follow-up frequency
Underlying issues for involving PHCs in continuity of care		Ambiguous role of PHCs Lack of integrated information system Lack of awareness of accessibility to continuity of care with PHCs	

### Patients’ experience of the lack of continuity of cancer care

#### Before admission: absence of a named doctor and a coordinator at PHCs

Patients described continuity of care as maintaining consistent contact with the primary physician from the tertiary hospital. The lack of understanding about the potential role of primary care as the starting point for health services, and their importance in ensuring continuity of care was evident from patients.

##### No existing referral strategy

The absence of a referral system for patients diagnosed or suspected of cancer created a significant barrier to continuity of care. Fourteen patients initially presented at PHCs, but none reported that PHC staff had introduced them to any pathway to a higher-level hospital.

*‘Six months ago, I had an abnormal result from a physical examination…no one advised me where to go.’ (M, 70, prostate cancer).*

*‘I do not remember which doctor I saw at the PHC, and I do not have their contact information either.’ (F, 70, breast cancer).*

Patients were therefore forced to navigate to tertiary hospitals by themselves. Ten participants relied on support from their family members for finding doctors in tertiary hospitals. Of these, seven patients also sought help from other cancer patients in similar situations. Another four patients went online to find out useful information.

This often led to delays in receiving care.

*‘I searched online to find which doctor specialized in treating the condition and had my daughter make inquiries as well……, it took over a month to gather the information. By the time I could proceed with the hospital examination, the cancer had metastasized.’ (M, 70, prostate cancer).*

##### Tertiary hospital as the first resource

The starting point in the patients’ treatment played a critical role in continuity of care. There is no gatekeeping policy to prevent them from going directly to tertiary hospitals.

*‘My wife and I did not know any doctors. I mean, for a big problem like this tumour, going to a tertiary hospital is a no-brainer. So, we just went ahead and booked an appointment with a chief doctor at the Oncology Hospital.’ (M, 71, gastric cancer).*

The trust participants placed in tertiary hospitals was evident, with all expressing confidence in the expertise of tertiary hospital staff.

#### During treatment: poor communication and discrepancies in treatments between providers

One-third of the patients had undergone surgery in Shanghai—a 2-hour drive from Nantong, and then received chemotherapy in Nantong. Communication between doctors from Shanghai and Nantong was virtually non-existent, jeopardising any opportunity for continuity of care. Moreover, according to the patients, chemotherapy regimens from different hospitals were frequently inconsistent with different treatment protocols, used in different hospitals.

##### Poor coordination during the treatment phase

The consistency of treatment protocols and continuity of information transfer was compromised when patients transitioned between healthcare settings.

*‘In Shanghai, I was prescribed just three chemotherapy sessions post-surgery. But in Nantong, the regimen expanded to 18 sessions.’ (F, 81, breast cancer).*

The scenario became more challenging for older patients who travelled to Nantong city from distant areas, especially when their children, who often served as their primary caregivers, lived far from them. The logistical challenges of transportation became a significant barrier, frequently leading to interrupted treatments.

*‘My son is working away and cannot take us to and from the hospital. So, we must go home and cannot carry on the treatment.’ (M, 68, colon cancer).*

##### Conflicts in decision making

Inconsistencies in treatment protocols caused patients to mistrust the doctors and led to hesitance in making decisions, thus affecting their adherence to treatment protocol. Patients were likely to refuse treatment and look for alternatives. Two patients who had been hospitalised more than once claimed to know more about their conditions than the tertiary doctors.

*‘(My doctor) asked me to have chemotherapy, but I declined. I was more concerned about quality of life than length. So, I took medicines and saw a traditional Chinese medicine (TCM) doctor.’ (F, 81, breast cancer).*

This might be because patients were sceptical about the medical advice from doctors who appeared not to understand them. Patients' trust in doctors is built when doctors demonstrate their understanding of the patients' health conditions, treatment histories and preferences. Furthermore, patients tend to trust doctors who can clearly explain the progression of their disease in an understandable way.

*‘He (the tertiary physician) knows me very well now. I reach out to him for any health concerns I have. He explains everything clearly, and I can easily understand all his suggestions.’ (M, 67, prostate cancer).*

##### Financial constraints

Costs of cancer treatment, especially for long-term treatment, emerged as an important barrier for continuity of care. Although each participant in this study was covered by medical insurance, none clearly understood the precise reimbursement rules, and all disclosed financial difficulties stemming from recurrent hospital admissions. This financial burden sometimes disrupted the consistent management of patients' conditions.

The out-of-pocket costs disproportionately impact less well-off patients. Patients undergoing repetitive and long-term treatments, who need to be hospitalised multiple times per year, face out-of-pocket costs averaging 80% of the actual costs, annually for medications and treatment. This creates a barrier to sustaining advised treatments and, consequently, continuity of care.

*‘Now I am spending too much on treatment, and I cannot claim much, only 20%. I spent more than 100,000 RMB (15,000 USD) last month.’ (F, 72, colon cancer).*

The integrity of medical advice was questioned by many, with about half of the participants suspecting profit motives. Moreover, patients doubted the financial interest of doctors when they were directed to buy medication from private pharmacies at their own expense.

*‘I had to buy Albumin in another designated pharmacy outside the hospital; I did not know whether there was any conflict of interest for the doctor.’ (M, 75, colon cancer).*

#### Post discharge and long-term care: dilemmas concerning patients’ needs and specialist-led follow-up care and rehabilitation

Every patient in our study received follow-up cancer care and rehabilitation led by tertiary hospital medical team members through regular outpatient check-ups and visits for adjuvant treatment. Twenty patients managed their long-term medical consultations by proactively texting their doctors to set up the next visit. Apart from three patients from the oncology hospital who occasionally received phone calls from their specialists, no other patients received any communication from their physicians.

The most significant factor affecting the continuity of care post-hospitalisation is that patients had no expectation that primary care could assume this role in follow-up and rehabilitation. When questioned about whether a patient would like to receive any follow-up services from PHC doctors, all but two patients said it was unnecessary.

*‘Why bother making contact with doctors from PHCs when I can just contact my surgeon?’ (M, 66, breast & lung cancer).*

##### Fragmented healthcare patterns

Since oncologists and surgeons focused solely on their own medical speciality, patients who had co-morbidities, particularly those vulnerable after cancer treatment, often struggled to manage the complexities of seeking and scheduling follow-up visits, monitoring, and tests with various specialists for their different ailments.

*‘I had to go back to the surgeon for changes of dressings, then went to the internal medicine department for chemotherapy treatment, then I had to make another appointment to get my cardiac drugs’ (M, 66, lung cancer).*

Consequently, the absence of coordinated aftercare from PHCs resulted in a fragmented treatment experience for the patient, disrupting the continuity of care essential for adequate recovery and management of their health. This emphasises the need for an integrated healthcare system.

##### Overburdened tertiary doctors

Almost all patients stated that seeing the same physician through treatment and recovery provided familiarity, reassurance and continuity. One patient mentioned that continuity of care over time meant that doctors could learn lessons from their own management of patients by seeing the outcomes of different treatments.

*‘When doctors were involved in long-term care, they got to know what works best for patient’s problems, and symptoms and this benefited other patients.’ (M, 75, colon cancer).*

An additional sensitive issue is that in some cases the cancer diagnosis was not fully disclosed to the patient, but rather to the family members. Maintaining a consistent relationship with the same physician can lead to a deep-seated understanding. A daughter of a patient mentioned this.

*‘I always told the doctor to communicate with me and the doctor knew that… Actually, my father is afraid of hearing any bad things…’ (M,70, prostate cancer).*

But providing such care under the current specialist-led healthcare system, it was unsustainable for tertiary doctors with large throughput of new patients to maintain interpersonal continuity post-hospitalisation.

Two-thirds of the participants expressed concerns that physicians were too busy, and they felt discouraged from presenting their concerns for fear of being seen as a burden or wasting the doctors' time.

*‘You cannot ask him. He has too many patients, many new patients everyday… If you required him to do such things, he would burn out! You need to be responsible for your health.’ (M, 70, lung cancer).*

##### Individualised follow-up frequency

Patients had diverse opinions about the frequency of follow-up. Three patients who received surgery, and considered their cancer as totally removed, thus not requiring further treatment. So, they were reluctant to contact doctors again. Another 10 patients highlighted the necessity for sustained engagement with their doctors, despite such needs potentially being deemed excessive according to the treatment guideline.

*‘I have a CT scan every 20 days now… If there is no problem, I will be reassured’ (M, 75, colon cancer).*

In the specialist-led care model, tertiary doctors lack the time to guide patients on appropriate follow-up care frequency. They also cannot provide personalised follow-up intervals to ensure continuity of care.

### Underlying issues for involving PHCs in continuity of care

#### Ambiguous role of PHCs

One of the fundamental issues of involving PHCs in continuity of care is that instead of having a collaborative relationship, tertiary hospitals and PHCs are financial competitive in attracting patients.

Patients described how tertiary hospital doctors were ‘possessive’ for hospitalised individuals.

*‘It has been two years since I underwent surgery, 55 times of hospitalization! And I have just been prescribed another six doses of chemotherapy. The chemotherapy treatment needed to be done here as an in-patient’ (F, 65, breast cancer).*

They also described PHC doctors occasionally provided treatments to cancer patients who were supposed to be served at higher-tier hospitals by more specialised doctors.

*‘I was mad at him (the PHC doctor) ……He directly started intravenous therapy and I thought I was going to die.’ (M, 75, colon cancer).*

Most patients expressed concerns about the limitations of PHC doctors’ skills especially when PHCs offered services beyond their expertise in cancer treatment. When patients observed discrepancies between the advice given by PHC doctors and by specialists in tertiary hospitals, this mistrust increased.

*‘It (PHC) was definitely not professional… When I came back from the oncology hospital and asked the doctors in township hospital for help, what they said was wrong.’ (M, 75, colon cancer).*

#### Lack of integrated information system

The absence of an integrated information system for sharing patient treatment information across tertiary hospitals, PHCs and other healthcare providers diminished the potential for incorporating PHCs into the continuity of care.

*‘I got all my blood work, liver and kidney checks, tumour marker tests, and a CT scan done just one week before getting admitted. When I showed up at the hospital this time, they made me do all those tests again from scratch. It was like they completely ignored what I did earlier I suspected they were having me undergo more tests just to make money’ (F, 76 breast cancer).*

The lack of integrated information forced patients to repeat their medical and treatment history to each new healthcare provider they encountered. Patients complained about the inconvenience, and cost.

*‘… yes, here (oncology hospital) is pretty far away from my home, but I was not up for seeing other doctors once more. Having to go over my whole medical situation again – it was very unpleasant.’ (M, 76, Gastric cancer).*

#### The lack of awareness about continuity of care with PHCs

Only patients with family members with previous personal connections with healthcare providers sustained interpersonal continuity with their physicians.

*‘... We (the patient and her surgeon) are like friends, I think. My son was his colleague before. For any problem, even small problems, I will go to him for help.’ (M, 66, breast & lung cancer).*

Compared to sustaining interpersonal continuity with tertiary hospital physicians, connecting with PHC doctors faced fewer constraints. PHC doctors are less burdened compared than their counterparts in larger hospitals, enabling PHCs to cultivate personalised caring relationships. However, patients did not seem to recognise (or chose to ignore) the potential role of PHCs in continuity of care, which is especially useful in addressing multimorbidity. Only two patients were satisfied with being followed up long term by their PHC doctors. They were retired workers living near their PHCs, with their children employed in distant locations.

*‘Now my situation is relatively stable. We usually go to HanYao (a PHC) to get TCM. The doctor there will help me deal with any problems I have at any time.’ (M, 66, lung cancer).*

*‘I have high blood pressure I have been treated at the community hospital for over 10 years. The staff there are always so patient and understanding. If I could just take my chemo drugs there, that would be amazing!’ (M, 71, colon cancer).*

## Discussion

This study provided insights into older patients’ experiences and perceptions of the lack of continuity of care during cancer treatment. There was no system in place to coordinate continuity of care between primary and tertiary facilities, despite efforts to promote an integrated healthcare system. Patients had virtually no awareness about the potential for good quality long term care at PHCs. This could be attributed to weaknesses in the government's health campaign, which does not alter ingrained attitudes about underlying mistrust towards PHCs.

The absence of primary care gatekeeping, enabling patients to directly present at tertiary level, along with minimal coordination between professionals at the different levels, are a major barrier to the role of PHCs in continuity of care. Following the WHO guidelines for continuity of care, our findings concluded that the absence of a referral policy, the lack of a care coordinator during treatment, the fragmented healthcare pattern and the unconnected information system are four key barriers to continuity of care, as shown in Figure 2.

None of the participants in this study mentioned that doctors recommended which healthcare providers they should visit after completing the current stage of treatment. This could be because many doctors from tertiary hospitals and PHCs have no knowledge of the integrated healthcare system policy. According to a survey conducted in Wuhan, China, only half of local PHC doctors were familiar with the integrated healthcare system [35]. In Zhejiang, a mentorship system between specialists and PHC doctors has been introduced to improve mutual understanding [36].

In this study, patients’ experience of interpersonal continuity with doctors is based on patients' trust related to their perceptions of the quality of care, rather than the actual quality of care. Previous studies have shown that repeated consultations with as few doctors lead to a trusting relationship [37]. However, this study indicated that while patients placed high value on having only one physician or one medical team throughout their treatment and follow-up this would be challenging given the high demands on tertiary doctors and their specialisation in cancer care. This challenge is present in many countries.

Findings from this study are consistent with existing evidence that integration, coordination and shared information between healthcare providers are essential in achieving high-quality continuity of care [38]. In our study participants complained about receiving contradictory information from healthcare providers at tertiary and primary levels, especially in complex cases. For cancer patients this may be crucial. It is well-recognised that continuity of care requires that tertiary hospitals and PHCs must have well-defined roles; PHC doctors also need to be in contact with tertiary doctors to access patients’ health records and receive detailed guidance on long-term treatment [39].

We found that healthcare financing plays an important role in ensuring continuity of care. As reflected by participants, tertiary doctors preferred to maintain direct contact with their patients, prescribe repeated tests, and were reluctant to refer patients to PHCs, even when they were more likely to receive personalised care. The fee-for-service payment system leads to perverse incentives: unnecessary overtreatment, including drugs, diagnostic tests and unnecessary hospitalisation, increasing the high costs associated with long-term cancer treatment to patients in tertiary hospitals [40].

PHCs currently have overlapping scopes of practice. Two of our interviewees received initial chemotherapy in PHCs. The ambiguous role of PHCs arises when the range of services offered is beyond their intrinsic scope [41]. Consequently, when the complexity of treatment exceeded the capacities of PHCs, it deepened patients’ mistrust, resulting in failure to return to PHCs for follow-up care. This aligns with several reviews regarding China’s healthcare system [42]. Prevention and screening of common chronic diseases and infectious diseases, which are the primary responsibilities of PHCs, are unprofitable. The financial challenge has led PHCs to shift their focus from prevention to outpatient care and, in some cases, inpatient care. As a result, PHCs struggle with balancing the need for more profit and their limited capabilities [43].

This study focused on the growing population of older patients with cancer. Our findings highlight two distinct characteristics of older patients which show the benefits of involving PHCs in continuity of care: firstly, older patients frequently encounter multimorbidity and face challenges with treatment protocols. Integrating PHCs into the referral and transition process, guided by oncologists, may facilitate a smoother exchange of patient treatment details and ensure longitudinal continuity of older cancer patients [44]. A cohort study from the Netherlands on the association between mortality for older patients and primary care continuity depicted poorer health outcomes related to missed opportunities for referral to a more appropriate professional with coordination of PHCs [45].

Secondly, social support for older cancer patients is predominantly provided by family members in China. Older patients are particularly dependent on their relatives for a range of needs, including approaching appropriate physicians, establishing communication with healthcare providers and arranging transportation for hospital visits. Those family members who have previous personal connections with doctors, tended to have easier access to physicians in tertiary hospitals and maintain closer relationships with them. On the contrary, these socioeconomic factors did not appear to influence the difficulty of establishing and keeping contact with PHC doctors. This finding demonstrated that the inherent advantages of PHCs, especially for older cancer patients, are proximity, convenient travel and familiarity with staff, providing a sense of trust and comfort [46].

### Strengths and limitations of this study

The potential for using PHCs for care coordination and continuity especially for older patients has become a focus of policy under the integrated healthcare system. To our knowledge, this is the first qualitative study to explore older cancer patients’ experience and perceptions of the lack of continuity of care in China,

Nantong is a relatively wealthy city with similar challenges in terms of healthcare resources utilisation, underuse of PHCs and overcrowded tertiary hospitals comparable to settings in other parts of China.

There are several limitations. First, we recruited participants from just two tertiary hospitals, and most patients were undergoing periodic treatment and regular follow-ups at tertiary facilities, and had not been treated at PHCs. Interviewing patients in the hospital where they are currently receiving treatment might introduce bias in their responses regarding the hospital's performance, as they might be inclined to respond more positively. Also, due to the long-term treatment as a characteristic of cancer care, reported experience may be subject to recall bias. Furthermore, patients tend to view experiences with PHCs as inferior to those at tertiary hospitals. This tendency can lead to social desirability bias, where patients conceal their PHC encounters to conform to perceived social expectations.

### Recommendations for future research and policy

In many high-income countries, established gatekeeping policies and referral systems provide systemic support necessary to ensure the implementation of continuity of care, though challenges persist. For example, in the UK, patients often complain about the reduced likelihood of seeing their preferred GP due to huge pressure on services [47]. Evidence from these countries is therefore not generalizable to China. In China using PHCs to achieve continuity of care is not feasible without gatekeeping policy. Meanwhile, there is increasing interest on enhancing the role of primary care in continuity of care in some low-and middle-income countries. For instance, community healthcare workers in Brazil are required to visit each family within the community at least once a month, providing health education, and managing minor health issues [48].

In China, integration mechanisms to improve coordination between tertiary hospitals and PHCs are needed. Currently, the continuity of care that patients receive depends on individual clinicians’ responsibility for patients’ long-term care. Hence, there is a need to understand healthcare providers' perspectives on continuity of care to develop uniform standards to ensure consistency and quality in the patient care continuum. For example, tertiary hospitals should establish formal connections with PHCs through comprehensive referral and follow-up systems under the ‘integrated healthcare system’; the medical alliances should establish electronic information platforms that aggregate all patient treatment records. Furthermore, practical evidence from intervention studies is required to evaluate the feasibility and effectiveness of these mechanisms and standards.

Existing evidence demonstrated that continuity of care at PHCs is associated with lower costs, especially for patients with complex conditions [49]. However, according to our findings, policies regarding the ‘integrated healthcare system’ and the potential financial and services benefits from regular PHCs visit are often overlooked or unknown to patients. PHCs have the potential to promote themselves by emphasising their delivery of affordable, high-quality, personalised and long-term care with the support of government and tertiary hospitals within the medical alliance. By effectively communicating this commitment, PHCs can better attract patients, especially the older patients with chronic conditions, including cancer. As positive experiences and success stories circulate, people may increasingly opt for PHCs for their healthcare needs.

Moreover, since integrating PHCs into continuity of care is a relatively new concept in China, there is limited evidence on both the extent and effectiveness of coordinated care provided by PHC doctors. Further exploration is needed to understand the continuity of care experience for patients in rural areas in China.

## Conclusion

The need for continuity of care at each stage of cancer care, including cancer detection, treatment, transfer and aftercare services, for older cancer patients is largely unaddressed and unmeasured in the current specialist-led cancer care model. The four dimensions of continuity of care are highly interrelated. Increasing interaction and trust between patients and PHCs through a systematic referral system from tertiary hospitals to PHCs after hospitalisation and guided and monitored by tertiary doctors. This could be a more sustainable and effective model of care to support greater continuity of cancer care and improved health outcomes.

## Supplementary Material

aa-24-0181-File004_afae213
