# Supplementary material for: Exploring the lack of continuity of care in older cancer patients under China's ‘integrated health system’ reform
Source: Age Ageing. 2024 Oct 7;53(10):afae213. doi: 10.1093/ageing/afae213 (PMC11457369; doi:10.1093/ageing/afae213)
Supplement: aa-24-0181-File004_afae213 [file aa-24-0181-file004_afae213.docx]

**Exploring the Lack of Continuity of Care in Older Cancer Patients under China's “Integrated Health System” Reform**

Supplementary Data

Contents:

[Appendix 1: Consolidated criteria for reporting qualitative studies (COREQ) checklist 2](#_Toc156900436)

[Appendix 2: Interview Guides 5](#_Toc156900437)

[Appendix 3: Characteristics of interview participants 8](#_Toc156900438)

### Appendix 1: Consolidated criteria for reporting qualitative studies (COREQ) checklist

| **No. Item** | **Guide questions/description** | **Details reported here or  in the main text of the paper** |
| --- | --- | --- |
| **Domain 1: Research team and reﬂexivity** | | |
| *Personal characteristics* | | |
| 1. Interviewer/ facilitator | Which author/s conducted the interview or focus group? | RL, JWG, and DXZ |
| 2. Credentials | What were the researcher’s credentials? E.g. PhD, MD | All authors have a background in research and medical practice/nurses.  Credentials: RL and JWG: PhD candidates.  DXZ: MSc |
| 3. Occupation | What was their occupation at the time of the study? | Researcher and nurse. |
| 4. Gender | Was the researcher male or female? | Both male and female researchers are in the research team. |
| 5. Experience and training | What experience or training did the researcher have? | All interviewers have previous qualitative research experience and received internal training in this qualitative research. |
| *Relationship with participants* | | |
| 6. Relationship established | Was a relationship established prior to study commencement? | No |
| 7. Participant knowledge of the interviewer | What did the participants know about the researcher? e.g. personal goals, reasons for doing the research | The participants received generic information about the study; no specific details about the researcher were provided. |
| 8. Interviewer characteristics | What characteristics were reported about the interviewer/ facilitator? e.g. Bias, assumptions, reasons and interests in the research topic | Various experiences and interest in continuity of care in the setting of primary care and older adults. |
| **Domain 2: Study design** | | |
| *Theoretical framework* | | |
| 9. Methodological orientation and theory | What methodological orientation was stated to underpin the study? e.g. grounded theory, discourse analysis, ethnography, phenomenology, content analysis | A combination of deductive and inductive thematic analysis was conducted to generate a thematic framework of patients’ experiences and perceptions of continuity of care. |
| *Participant selection* | | |
| 10. Sampling | How were participants selected? e.g. purposive, convenience, consecutive, snowball | A purposive sampling method was used to obtain a sample of patients by age, sex, and cancer type. The target population was patients aged 65 years and over, following in-hospital treatment for cancer, including chemotherapy, radiotherapy, and surgical treatment. The types of cancer were lung, gastric, colon, liver, breast, and prostate, the most common cancers in older adults in China. |
| 11. Method of approach | How were participants approached? e.g. face-to-face, telephone, mail, email | Participants were identified from a questionnaire survey regarding satisfaction of healthcare services, administered to all cancer patients hospitalized from March to December 2022 in Nantong Oncology Hospital and the cancer department of the Affiliated Hospital of Nantong University. At the end of the questionnaire, patients were asked if they were willing to participate in an interview about their experience and understanding of continuity of care and to provide their contact details.  We had planned to conduct all interviews face-to-face. However, the restrictions of COVID-19 made this impossible. Therefore, we conducted interviews with 17 patients by phone at their home and 12 patients face-to-face on the ward, just prior to their discharge, between July 2022 and January 2023. Interviews lasted 40 minutes on average. |
| 12. Sample size | How many participants were in the study? | 29 participants. |
| 13. Non-participation | How many people refused to participate or dropped out? Reasons? | As participants were recruited via expression of willingness at the end of the questionnaire, no exact numbers or reasons are available. |
| *Setting* | | |
| 14. Setting of data collection | Where was the data collected? e.g. home, clinic, workplace | We called 17 patients by phone at their home. The other 12 patients were met face-to-face on the ward. |
| 15. Presence of non-participants | Was anyone else present besides the participants and researchers? | In 7 interviews, patients’ family members were present. |
| 16. Description of sample | What are the important characteristics of the sample? *e.g. demographic data, date* | Reported in Results and Appendix 3. |
| *Data collection* | | |
| 17. Interview guide | Were questions, prompts, guides provided by the authors? Was it pilot tested? | Interview guides (Appendix 2) were structured following the WHO continuity and coordination of care framework mentioned above. We started analysing data after the first three interviews were conducted, so the qualitative process was iterative, with responses informing the subsequent interviews; new questions were added, and some questions were simplified. |
| 18. Repeat interviews | Were repeat interviews carried out? If yes, how many? | No. |
| 19. Audio/visual recording | Did the research use audio or visual recording to collect the data? | All interviews were audio-recorded and transcribed verbatim. |
| 20. Field notes | Were ﬁeld notes made during and/or after the interview or focus group? | Interviewers made summaries after each interview. |
| 21. Duration | What was the duration of the interviews or focus group? | Each interview lasted around 40 minutes. |
| 22. Data saturation | Was data saturation discussed? | Sampling was continued until saturation was expected to be reached, based on discussion in the research team. In the last performed interviews, no new information came up. |
| 23. Transcripts returned | Were transcripts returned to participants for comment and/or correction? | No. |
| **Domain 3: Analysis and ﬁndings** | | |
| *Data analysis* | | |
| 24. Number of data coders | How many data coders coded the data? | Twelve interviews, from each stakeholder in each country, were independently coded by AM and EH, to form a preliminary codebook. AM, EH, and WG applied this codebook on the remaining interviews. |
| 25. Description of the coding tree | Did authors provide a description of the coding tree? | No. |
| 26. Derivation of themes | Were themes identiﬁed in advance or derived from the data? | Initially themes were derived from the data. |
| 27. Software | What software, if applicable, was used to manage the data? | QSR NVIVO 12 software |
| 28. Participant checking | Did participants provide feedback on the ﬁndings? | No. |
| *Reporting* | | |
| 29. Quotations presented | Were participant quotations presented to illustrate the themes/ﬁndings? Was each quotation identiﬁed? e.g. participant number | Reported in Results. |
| 30. Data and ﬁndings consistent | Was there consistency between the data presented and the ﬁndings? | Reported in Results. |
| 31. Clarity of major themes | Were major themes clearly presented in the ﬁndings? | Reported in Results. |
| 32. Clarity of minor themes | Is there a description of diverse cases or discussion of minor themes? | Variation between participants is described in the themes. |

### Appendix 2: Interview Guides

| - **Socio - Demographic information**  1. Age 2. Medical History 3. Residential place  - **Cancer related:**  1. How do you detect cancer?   (When you came to the cancer hospital to see a doctor, did you find out that you were sick through a physical examination, or did you go there yourself when you were not feeling well and had symptoms? Or recommended by doctors in other hospitals? Do you still have contact with your previous doctor?)   - **Information continuity**  1. Have you checked again at the cancer hospital? 2. How long has it been since your surgery? Did you feel any discomfort after the surgery? Did you do any other treatments after the surgery? (Chemotherapy)  - **Interpersonal continuity**  1. Who did you communicate with during your recovery in the hospital after surgery?   (Are the outpatient, surgery, and bed-in-charge doctors the same person? Apart from the doctor, are there nurses, nutritionists, and nurses involved? During the hospitalization, are there any cases where no one can be found? Does anyone care? After discharge, these people will be there later. communicate?)   1. How do you feel about your recovery?   (urination, incision, appetite, whether there is regular review) What kind of recovery state do you want to achieve?)   - **Management continuity**  1. Did you receive a discharge letter upon discharge?   (There are some summaries and care plans for your condition, medication, personal health management, etc.)   1. Have you read it? 2. What do you get from it? 3. Do you understand, do you think it will help you in your life? 4. What problem did it solve for you? 5. In addition to this report, has the doctor verbally told you to tell you about your life guidance? 6. Do you think this discharge letter considers your personal needs? 7. How do you think the discharge report can be improved? 8. Before leaving the hospital, did you have any concerns? Were you willing to leave the hospital? 9. Did your doctor tell you about your follow-up plan when you were discharged from the hospital?   (There is also risk assessment, that is, in the future life, what problems may arise, how to protect themselves, and how to solve them? How was your quality of life after you were discharged from the hospital? Are you afraid of relapse again.)   1. Did the doctor make an appointment with you for a re-examination when you were discharged from the hospital? Are you all on time? (If not, why) Seeing the distance, was it inconvenient. 2. After being discharged from the hospital, apart from the follow-up consultation, have the doctor contacted you and how? 3. Can you talk to us about the follow-up services you have actually received since you were discharged from the hospital?   (Did you leave a number when you were discharged from the hospital, did the doctor help with referral, help with information transfer and tracking with doctors in community hospitals, did they communicate or contact the doctor)   1. Do you remember the first visit? 2. Did the doctor call to ask you or did you go to the hospital for follow-up? 3. Do you think it's too early or too late for your first follow-up visit? 4. How did you feel about your recovery before your first visit? 5. What did you do during this period?   (Drugs, diet, bowel movements, exercise, I see that you are still very careful in your life. Are these all taught by doctors? Or do you do it yourself after finding information)   - **Longitudinal continuity**  1. How many times have you been followed up so far?   (Do you check every time? Are the exams scheduled or tailored to your medical condition - patients express concerns, have symptoms)   1. The form (outpatient review, phone calls, text messages, online) and content of all follow-up visits you received   (postoperative wounds, complications, side effects, recurrence, medication guidance, compliance, fall prevention, lifestyle such as diet, psychological care, cancer pain management)   1. Is it a phone call or WeChat?   (when you have a problem, have you contacted the doctor, and is there a situation where you don’t know who to ask?)   1. Do you think follow-up will help you in your recovery? What do you suggest? 2. Are you satisfied with your current follow-up? Did you meet your expectations? Did it meet your needs?   (Does each follow-up feel that the problem is adequately resolved, or is the doctor patient? Is it timely)  ①Dissatisfied, which ones are dissatisfied, and how do you want to improve?  ② Satisfied, where is it good, where can it be better?  ③Are you anxious? Worried? Is there anything you need?   1. Who attended the follow-up? 2. Is the doctor the same person at the follow-up visit? 3. Has the attending doctor called you? 4. Do you think follow-up is important for a person? 5. What kind of help do you want most in your post-operative recovery? 6. Do you think the frequency of follow-up is more or less? How long do you want doctors to know about your health and care about you? 7. During the follow-up, did your feedback get feedback? Does the doctor know about your recent physical condition? 8. Do you think follow-up is important? What do you think is more important during follow-up?   (Information itself? Communication and interaction? Monitoring? Adjustment? Timely symptom or problem detection? Self-management capabilities)   1. Did you have any physical problems after being discharged from the hospital? What about emotions? Do you need help in particular? 2. Do you have a patient group?  - **Management continuity and attitude of primary healthcare**  1. Do you usually go to a community hospital?   (Have you been there since you were discharged from the hospital?)  ①Yes, for what purpose? Does anyone at the community hospital know your medical history? Do you think they should know? Did it cause you any trouble?  ②No, do you think community doctors can monitor your health status if they know your medical history  (identify signs of early recurrence, medication, diet, etc., interact with you, and give health education)  ③Because doctors in large hospitals may be in charge of many patients, they cannot be very detailed; community hospitals are close to home; do you feel better?   1. How would you feel if community hospitals could participate in your aftercare and track your recovery? Do you think this is possible? What are your concerns about this? |
| --- |

### Appendix 3: Characteristics of interview participants

| Number of Patients | Age | gender | Hospital ^a^ | Cancer Type | Stage of treatment | Interview with family members |
| --- | --- | --- | --- | --- | --- | --- |
| Patient 1 | 67 | Male | 1 | Prostate | Routine chemotherapy | No |
| Patient 2 | 70 | Male | 1 | Lung | The first chemotherapy |  |
| Patient 3 | 81 | Female | 2 | Breast | Surgery of recurrence. First detection of cancer 2015 | No |
| Patient 4 | 65 | Female | 1 | Breast | Discharged one week after surgery of recurrence. First detection of cancer 2018 | No |
| Patient 5 | 66 | Female | 1 | Breast | Recovery phase with oral drugs | No |
| Patient 6 | 76 | Female | 1 | Breast | Routine chemotherapy | No |
| Patient 7 | 70 | Female | 2 | Breast | A week after surgery | Daughter |
| Patient 8 | 71 | Male | 1 | Colon | Two weeks after surgery | No |
| Patient 9 | 65 | Male | 1 | Colon | Waiting for surgery in hospital for one week | No |
| Patient 10 | 75 | Male | 1 | Colon | Routine chemotherapy | No |
| Patient 11 | 78 | Female | 2 | Lung | Four months after surgery | No |
| Patient 12 | 79 | Female | 2 | Breast | At the day of surgery discharge | Daughter |
| Patient 13 | 69 | Female | 2 | Lung | Routine chemotherapy and radiotherapy | No |
| Patient14 | 82 | Female | 2 | Liver | Oral medicine | Daughter |
| Patient 15 | 68 | Male | 1 | Colon | Routine chemotherapy | Wife |
| Patient 16 | 70 | Male | 1 | Prostate | Oral drugs | No |
| Patient 17 | 65 | Female | 2 | Breast | Oral drugs | No |
| Patient 18 | 65 | Female | 1 | Breast | Routine chemotherapy | No |
| Patient 19 | 71 | Female | 1 | Breast | Oral drugs | Daughter |
| Patient 20 | 79 | Female | 1 | Gastric | Routine chemotherapy and refuse to surgery | Daughter |
| Patient 21 | 66 | Male | 2 | Breast + Lung | Breast cancer 10 years ago now chemotherapy for lung caner | No |
| Patient 22 | 76 | Male | 1 | Gastric | Routine chemotherapy | Son |
| Patient 23 | 79 | Male | 1 | Gastric | Routine chemotherapy | Son |
| Patient 24 | 71 | Male | 2 | Gastric | Routine chemotherapy | Son |
| Patient 25 | 75 | Male | 1 | Colon | Routine chemotherapy | No |
| Patient 26 | 68 | Male | 2 | Lung | Routine chemotherapy | No |
| Patient 27 | 72 | Female | 1 | Colon | Routine chemotherapy | No |
| Patient 28 | 66 | Male | 2 | Lung | Routine chemotherapy | Wife |
| Patient 29 | 67 | Male | 1 | Prostate | Complication of surgery and stay in hospital for one month | Wife |

a: Hospitalized in Oncology hospital: 1; Hospitalized in the Affiliated Hospital of Nantong University: 2
